# Supplementary material for: Efficacy and safety of extracorporeal shockwave therapy for the treatment of chronic non-bacterial prostatitis: A systematic review and meta-analysis
Source: PLoS One. 2020 Dec 28;15(12):e0244295. doi: 10.1371/journal.pone.0244295 (PMC7769278; doi:10.1371/journal.pone.0244295)
Supplement: Attachment — Submitted filename: Response to Reviewers.docx [file pone.0244295.s002.docx]

**Response to Reviewers**

Submission ID: PONE-D-20-28346

Title	: Efficacy and safety of extracorporeal shockwave therapy for the treatment of chronic non-bacterial prostatitis: a systematic review and meta-analysis

Dear Editor, Reviewer 1, and Ms./Mrs. Maria de Lourdes Pereira

Thank you for taking the time to read our article and provide feedback on improving it. We have made quite a lot of changes in our article, and these are some point we would like to enclose regarding the comment that editor and reviewers have given:

**Editor:**

“*Please ensure that your manuscript meets PLOS ONE's style requirements, including those for file naming. The PLOS ONE style templates can be found at…*”

According to the template given, we have made an update regarding our article style, including the file naming.

“*Please include captions for your Supporting Information files at the end of your manuscript, and update any in-text citations to match accordingly. Please see our Supporting Information guidelines for more information:* [*http://journals.plos.org/plosone/s/supporting-information*](http://journals.plos.org/plosone/s/supporting-information)”

Thank you for the suggestion given. We thought that the pictures and tables in our main article are sufficient to support our article; thus, we neither consider adding additional figures nor tables as Supporting Information.

“*Thank you for submitting the above manuscript to PLOS ONE. During our internal evaluation of the manuscript, we found significant text overlap between your submission and the following previously published works.*”

We have updated the sections that overlap with the previously published article. We apologize for this negligence; we will try to be more thorough before submitting the article.

**Reviewer 1:**

*“Very few studies have been considered to support the final assumptions concerning the consensus on a dose of ESWT.”*

There are only a few studies available that discuss ESWT dosage. Thus, we also include this within the limitations of our study. The dose-response gradient cannot be assessed given that all included studies had the same dose of ESWT.

*“The authors should also take some caution regarding Pain Domain conclusions.”*

The pain domain after 12 weeks was the only outcome that had significant heterogeneity (92%). Therefore, we tried to analyze the effect size using the random-effects model. In this analysis, we found significantly lower results in the intervention group. However, this high heterogeneity may lead to less precise results, especially with the small number of studies in our meta-analysis. Thus, we also include this within the limitations of our meta-analysis.

**Reviewer 2:**

*“In general, the English language should be improved and errors corrected.”*

We would like to thank you for the feedback given. We have made several changes in our article. We also have tried our best to improve grammatical stuff in our article. We are looking forward for any comment regarding these revisions.

*“Abstract - Please detail some search engines used in this MS and period of study*

*Keywords: Please add “Chronic pelvic pain syndrome”*

Corrected

*“Introduction - This section must be more robust, making an upto date of the literature. In addition, please detail and rephrase aims of study, since some papers are available on ESWT treatment.”*

Changed

*“Results: Is this analysis based on three studies only? Which parameters were used to qualify two of those studies as good quality? Table 3 is expendable, since data are included in Table 4. Please correct errors in the footer of Table 4.”*

This analysis has made based on three studies available only. We used Jadad scale and Cochrane Risk Bias tools to assess the quality of these studies. We have expended the Table 3, and add a row in Table 4 informing the quality of study (Jadad scale score).

*“Discussion: Please start discussion section with the paragraph: “………*

*Please discuss other therapeutic startegies since a number of studies suggest a multimodal therapeutic approach.”*

Corrected. We also have added some discussion regarding the multimodal therapeutic strategies.
