# Supplementary material for: Efficacy and safety of extracorporeal shockwave therapy for the treatment of chronic non-bacterial prostatitis: A systematic review and meta-analysis
Source: PLoS One. 2020 Dec 28;15(12):e0244295. doi: 10.1371/journal.pone.0244295 (PMC7769278; doi:10.1371/journal.pone.0244295)
Supplement: Attachment — Submitted filename: Response to Reviewers.docx [file pone.0244295.s003.docx]

**Response to Reviewers**

Submission ID: PONE-D-20-28346

Title	: Efficacy and safety of extracorporeal shockwave therapy for the treatment of chronic non-bacterial prostatitis: a systematic review and meta-analysis

Dear Editor, Reviewer 1, and Ms./Mrs. Maria de Lourdes Pereira

Thank you for taking the time to read our article and provide feedback on improving it. We have made quite a lot of changes in our article, and these are some point we would like to enclose regarding the comment that editor and reviewers have given:

**Reviewer 1:**

*“The revised version has been improved. From my point of view the explanation regarding pain domain should be in the manuscript.”*

Thank you for the feedback has been given. We have added a brief additional explanation regarding the pain domain that we have found in this study, specifically in the discussion section.
